# Supplementary material for: CAF08 adjuvant enables single dose protection against respiratory syncytial virus infection in murine newborns
Source: Nat Commun. 2022 Aug 2;13:4234. doi: 10.1038/s41467-022-31709-2 (PMC9346114; doi:10.1038/s41467-022-31709-2)
Supplement: Supplementary file 1 — Supplementary Information [file 41467_2022_31709_MOESM1_ESM.pdf]

Supplementary Figure 1.

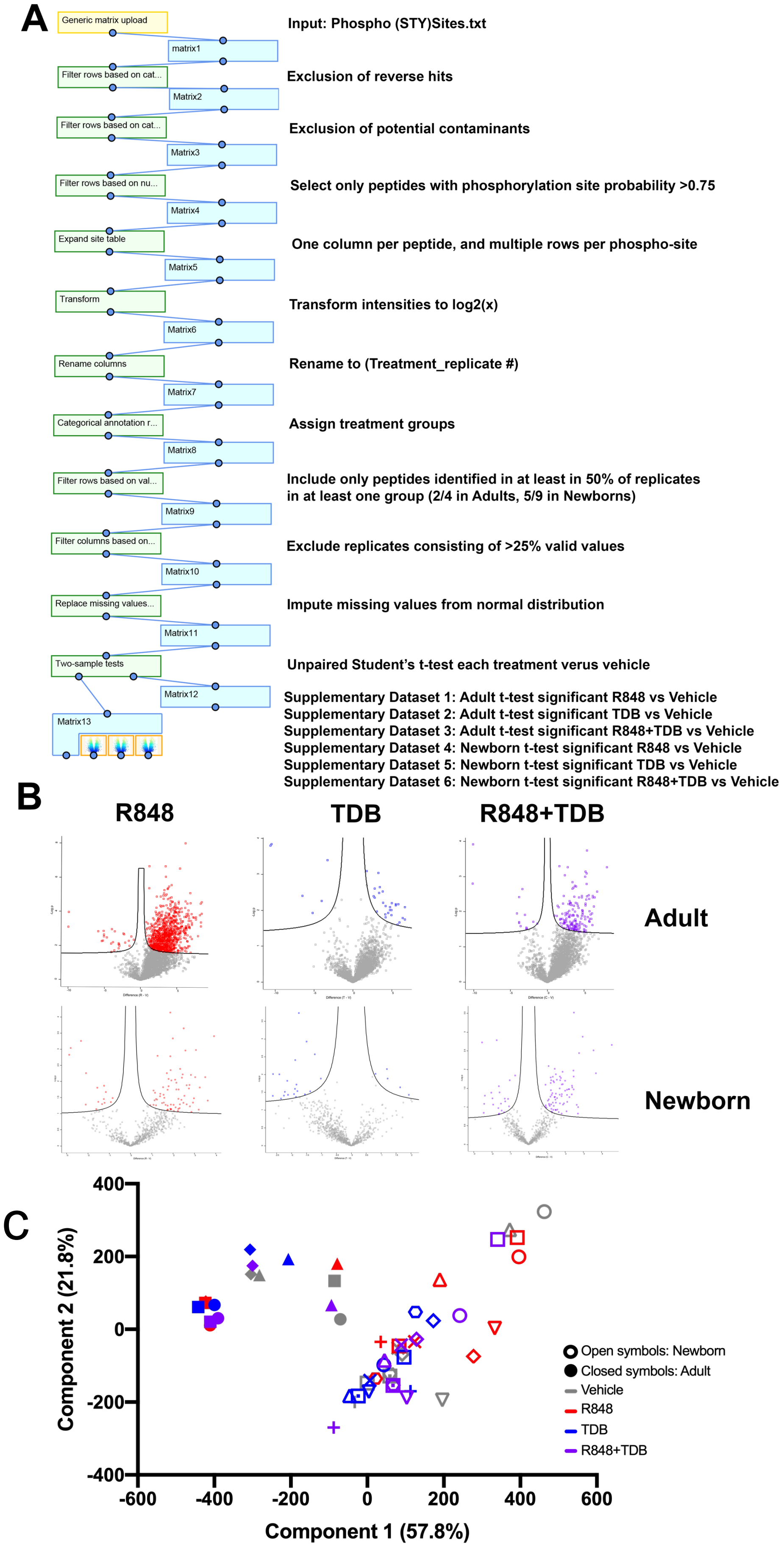

Supplementary Figure 1. Phosphopeptide quantification.

Newborn and adult (n=9 and n=4, respectively) MoDCs were stimulated with 50 µM R848, 100 µg/ml TDB, combination, or vehicle control for 30 minutes. Phosphopeptides were enriched from cell lysates and quantified by mass spectrometry as described in the Methods. **A)** Peptide intensities were analyzed using Perseus. The flow diagram depicts the analysis pipeline. T-test significant peptide data were exported to excel files, all of which are available as supplementary data tables to ensure reproducibility of our results. B) Volcano plots indicate changes upon treatment in phosphopeptide intensity. X-axes represent Log2-fold changes over vehicle treatment condition, Y-axes represent -Log10 (p value, unpaired two-tailed Student's t-test with a permutation-based fold-change threshold cutoff). A weighted p-value was applied with FDR 0.05 and σ=0.2. **C)** A Principal Component Analysis (PCA) model was constructed to reduce the dimensionality of the datasets by creating new uncorrelated variables that successively maximize variance. In the two-dimensional space of principal components (PCs) PC1 versus PC2, an apparent separation can be observed between newborn and adult samples. Minimal clustering of samples by treatment condition was observed.

Supplementary Figure 2.

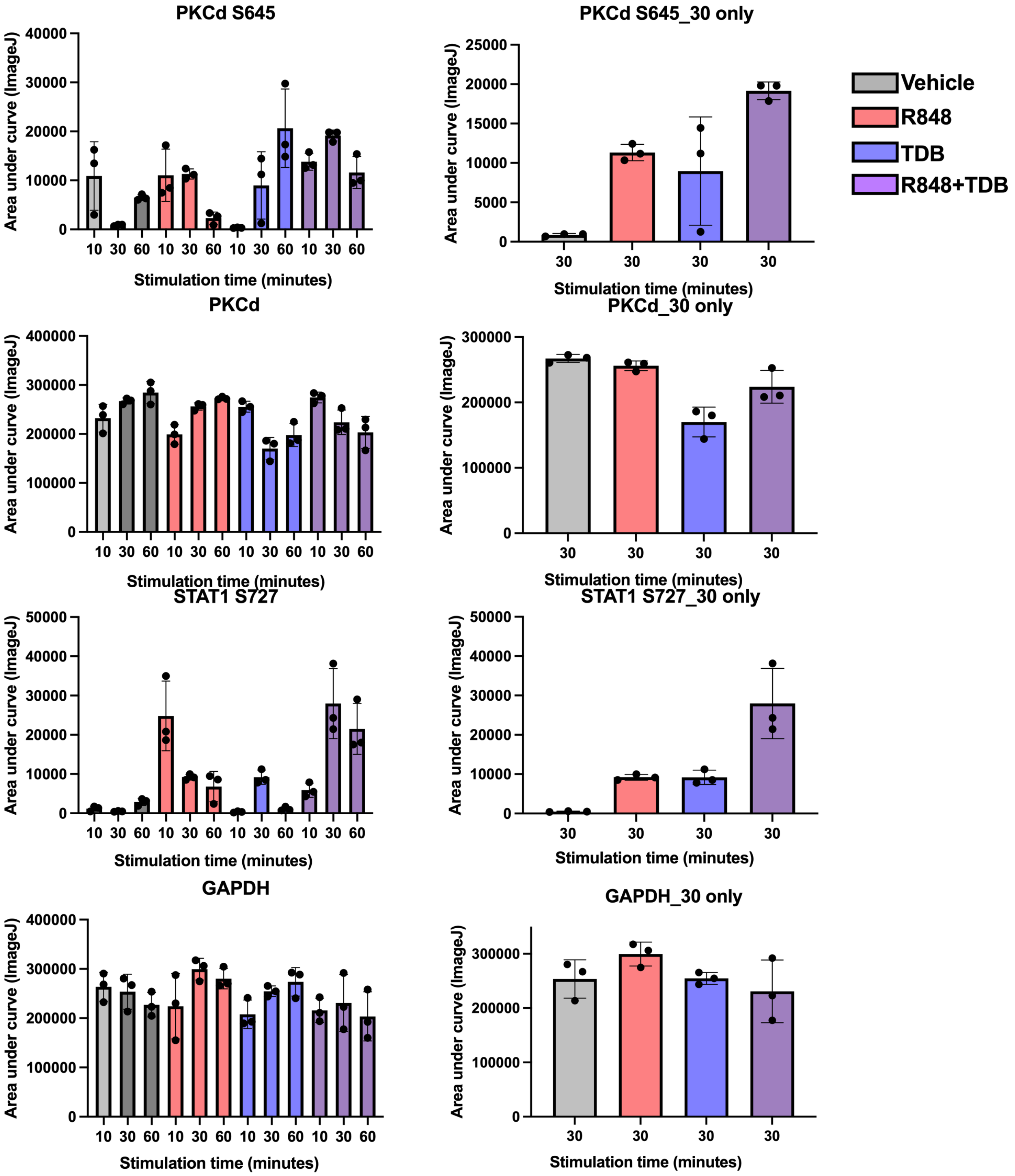

Supplementary Figure 2. Quantification of phosphorylated PKC-d and STAT1 by western blotting.

Protein abundance of PKC-d, phosphorylated at Serine 645, and STAT1, phosphorylated at Serine 727, was determined by western blotting, as seen in Figure 1E. ImageJ software was used to quantify relative signal intensity across three replicate experiments.

Panels on the right show relative intensities for 30-minute bands only, for comparison to the mass spectrometry results shown in Figure 1C-D.

Supplementary Figure 3.

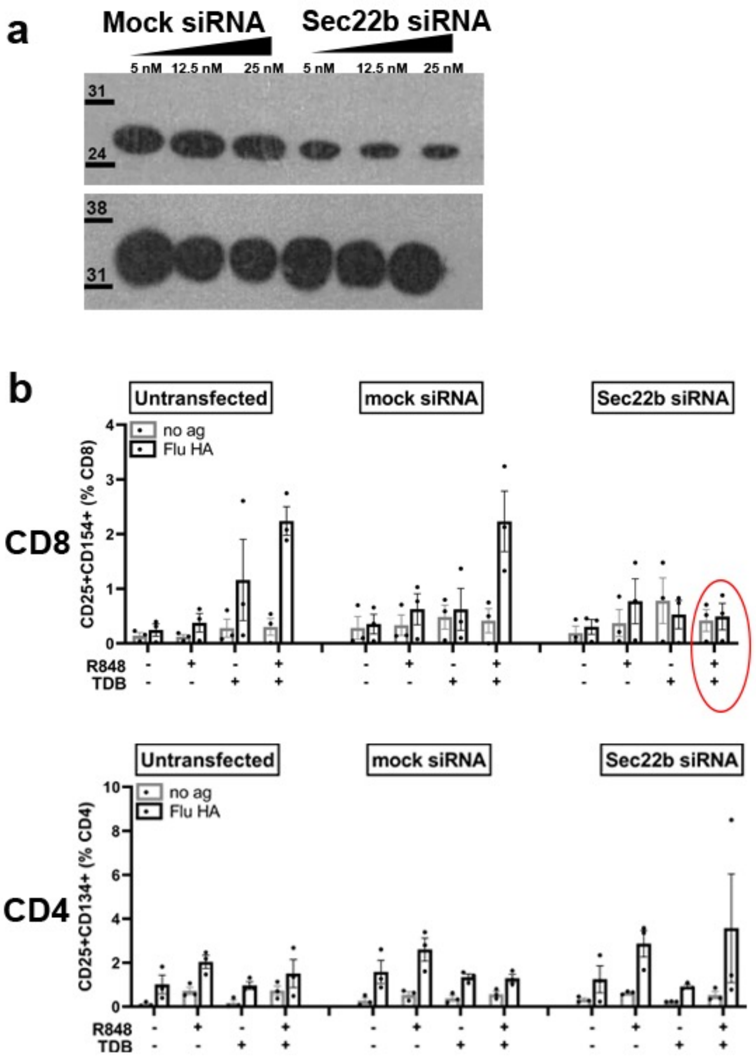

Supplementary Figure 3. siRNA-mediated inhibition of Sec22B abrogates R848+TDB-induced CD8 T cell activation. Protein levels of a key signaling adapter identified in our phospho-proteomics platform, Sec22b, were diminished in moDCs using a pool of 4 siRNAs (~85% reduction by western blot **(A)**). Reduction of Sec22b using siRNA, but not a pool of 4 non-targeting siRNAs (Mock siRNA), resulted in lack of activation of autologous HA-specific $CD8^+$ T cells **(B)**, but not of autologous HA-specific $CD4^+$ T cells **(C)**. (A-C: N=3 adult participants.)

Supplementary Figure 4.

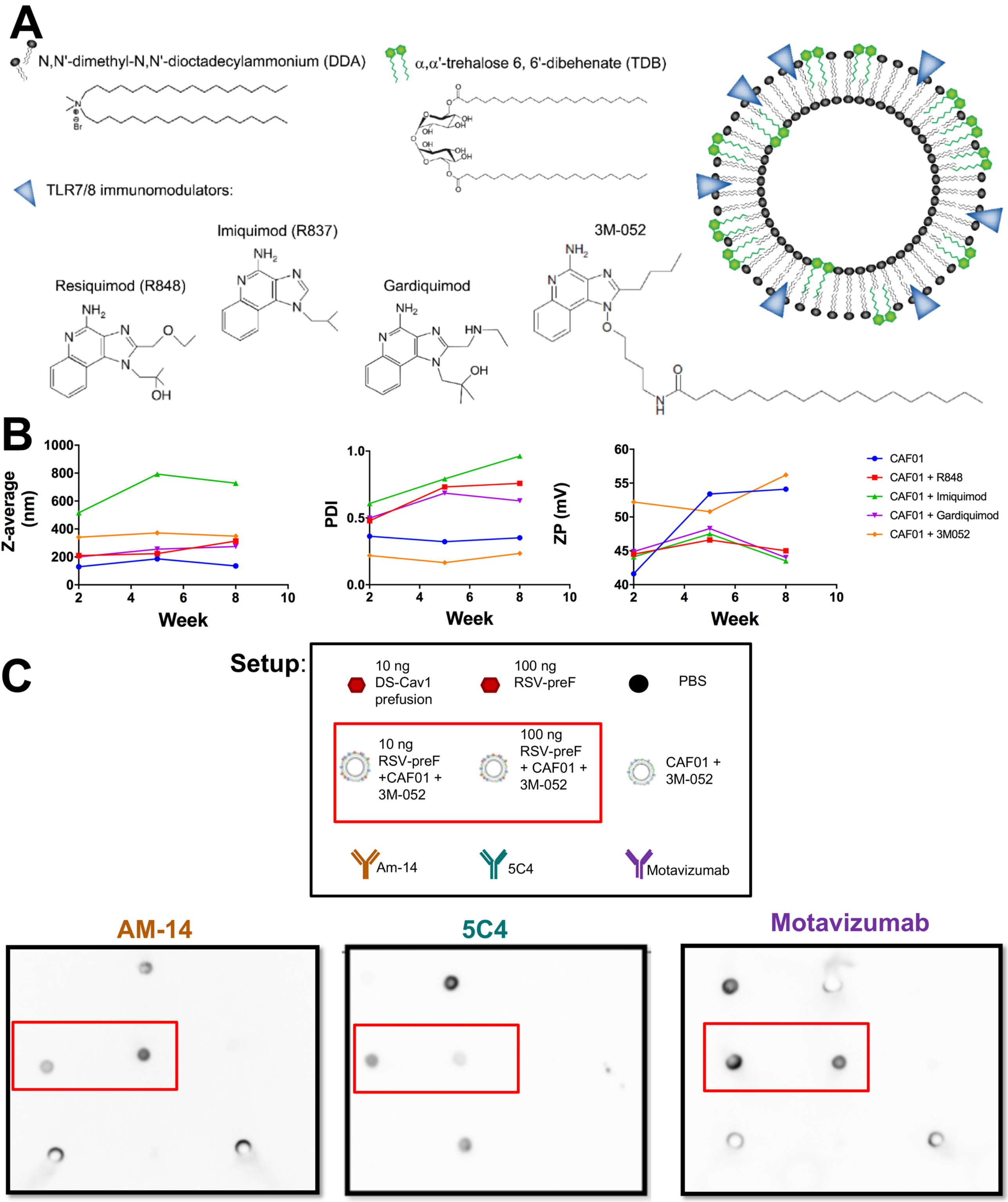

Supplementary Figure 4. Formulation optimization.

Different R848 congeners **(A)** were tested for incorporation into DDA and CAF01 liposomes. Liposomal stability was assessed by multiple dynamic light scattering measurements over 8 weeks **(B)**. Dot Blot of spotted formulations show the availability of three different antigenic epitopes on the pre-F protein after formulation, using monoclonal antibodies against pre-F as indicated **(C)**.

Supplementary Figure 5.

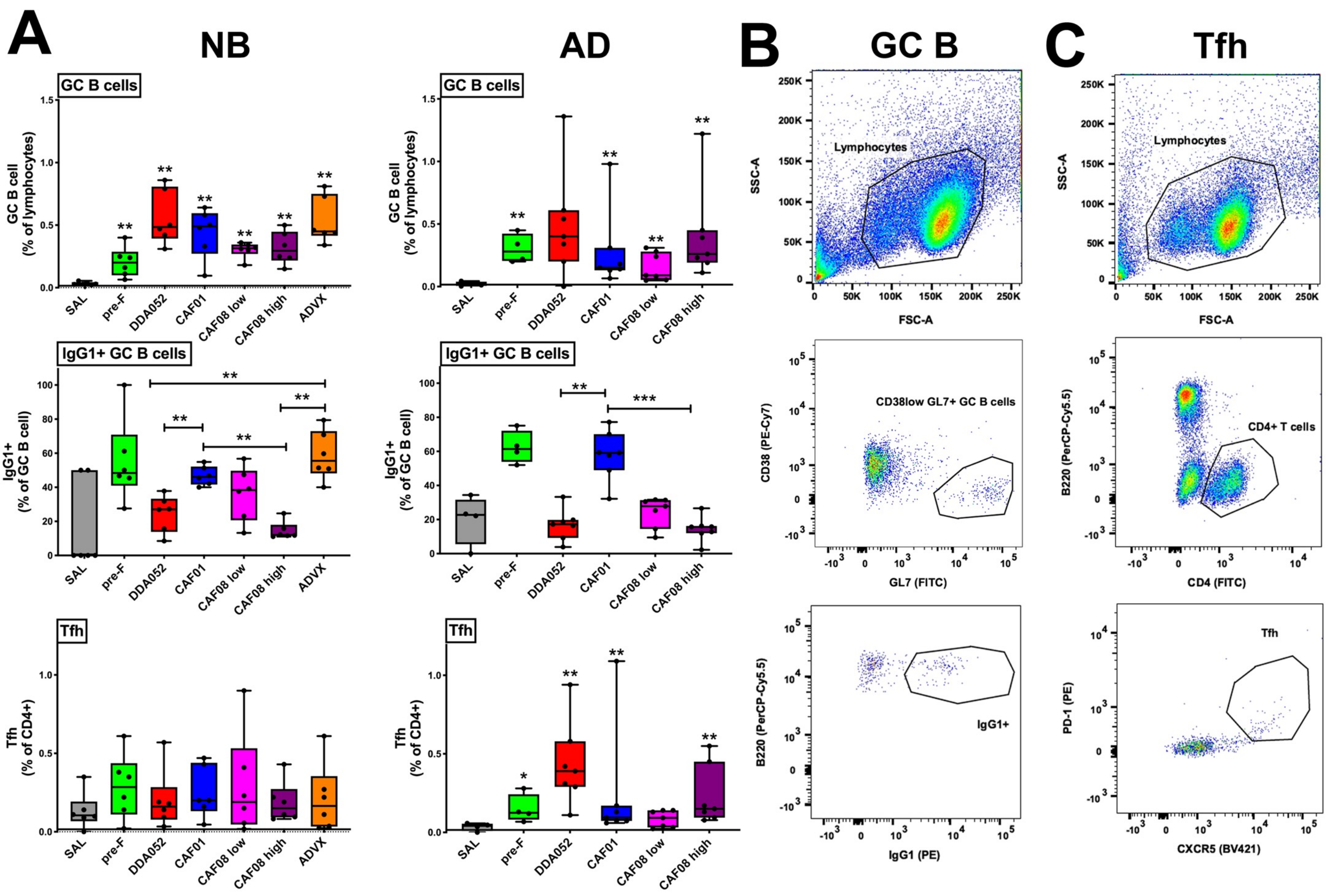

Supplementary Figure 5. Combination adjuvant-containing liposomes promote formation of germinal center IgG1- B cells and generation of Tfh cells.

Brachial or inguinal lymph node cell suspensions from immunized animals were analyzed phenotypically by flow cytometry. Immunization with formulations containing pre-F increased B220+GL7+CD38lo germinal center (GC) B cells in newborn and adult mice **(A)**. A reduction in IgG1+ GC B cells was noted after treatment with 3M-052-containing formulations, consistent with serology results in Figure 3, indicating isotype switching to IgG2a/c in those groups. An increase in B220-CD4+PD-1hiCXCR5+ T follicular helper (Tfh) cells occurred in adult mice immunized with DDA052, CAF01 or CAF08b_h formulations, but not in newborn mice. (n=6 newborn mice/group or 4 adult mice/group, 2-way ANOVA with Tukey post-hoc test, error bars indicate mean+SEM. *:p<0.05, **:p<0.01, ***:P<0.001). Representative gating is shown for GC B cells **(B)** and Tfh cells **(C)**.

Supplementary Figure 6.

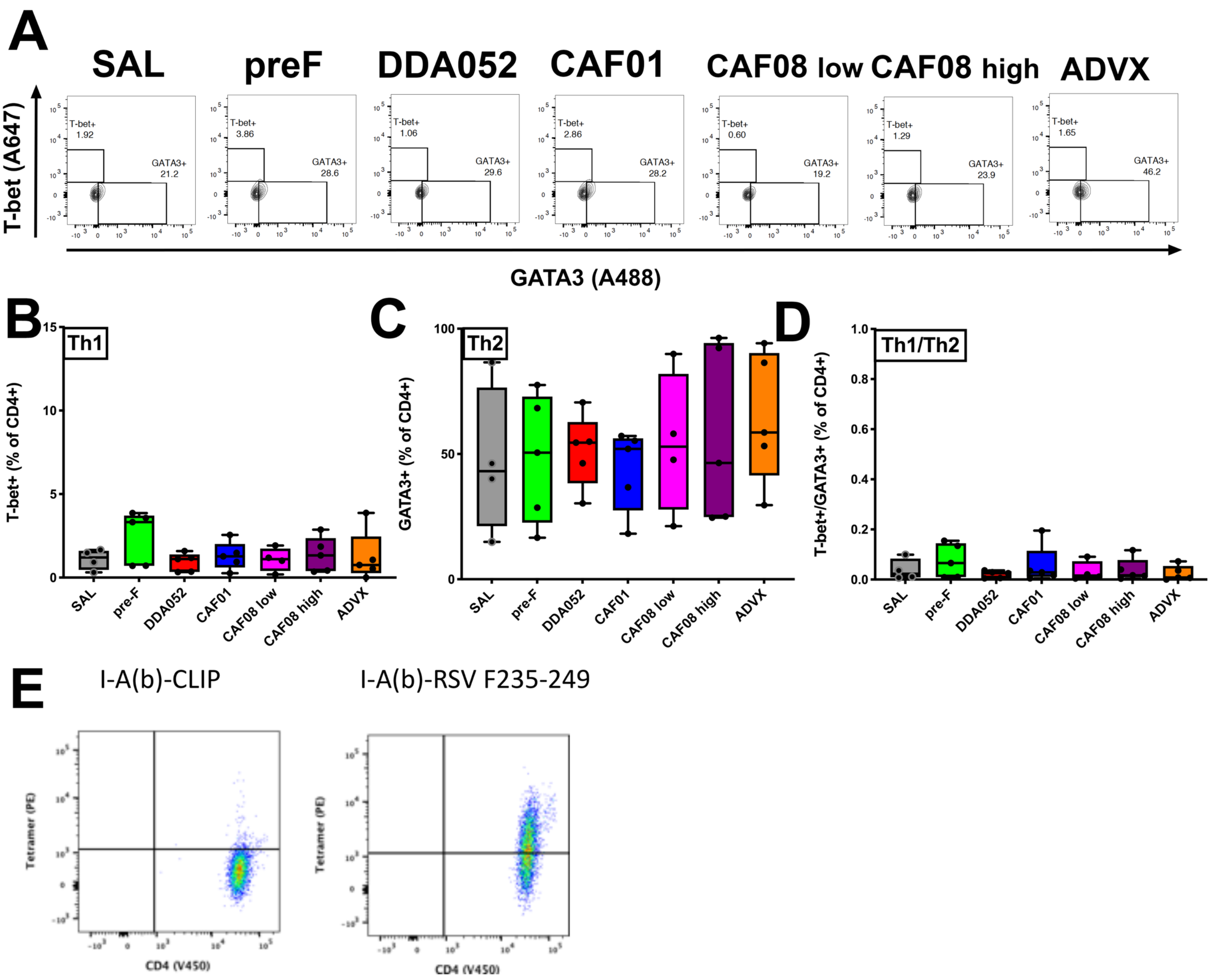

Supplementary Figure 6. Tetramer-negative T cell phenotype and tetramer-positive clone development.

Spleen and lymph node cell suspensions were stained for flow cytometry with a pre-F -specific MHC II tetramer (see figure 4) and stained for intracellular presence of Th1 transcription factor T-bet or Th2 transcription factor GATA3. Representative histograms of tetramer-negative cells are shown in panel **(A).** Quantification of T-bet+ Th1 cells **(B)** and GATA3+ Th2 cells **(C)**, revealed that The phenotype of tetramer-negative cells was predominantly Th2-skewed in all treatment groups, in accordance with the neonatal phenotype **(D)**. This indicated that Th1 induction by CAF08 was antigen-specific and did not result in systemic changes in Th1/Th2 balance.**.** LN cells from a CAF08-immunized mouse were stained with the tetramer and tetramer-positive cells were single-cell sorted and expanded using anti-CD3/28 beads. This resulted in the generation of a Tetramer-positive T cell clone **(E)**. **B-D**: n=5 newborn mice/group.

Supplementary Figure 7.

A

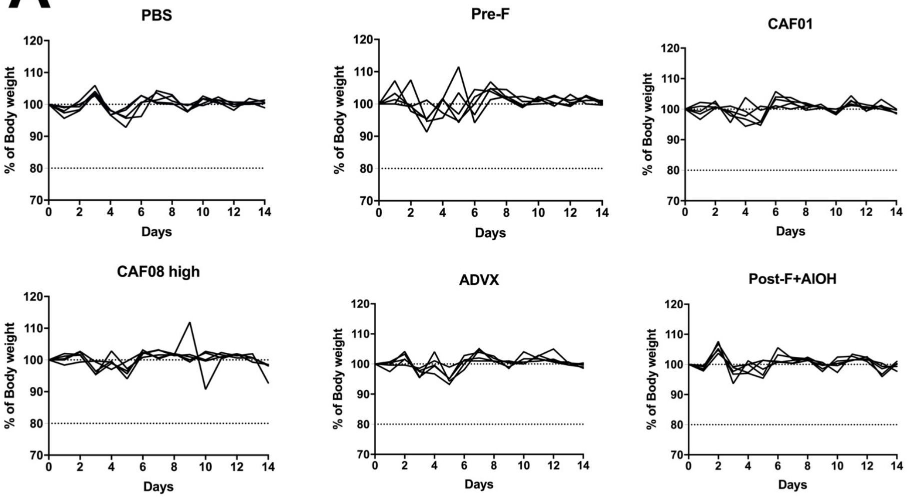

B

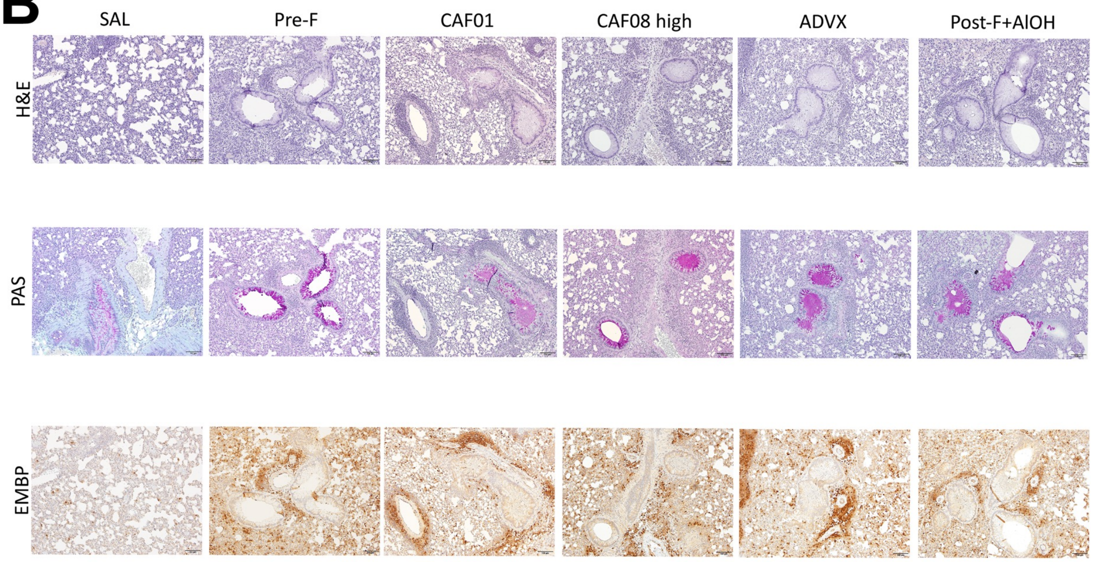

C

Sample ID	H&E	PAS/Mucin	EMBP
Gp1 (sample 1) Saline	-	-/-	+/-
Gp1 (sample 2) Saline	+/-	G/-	+/-
**Gp1 (sample 3) Saline**	+/-	**G/-**	+/-
Gp2 (sample 1) Pre-F	+	+ G/M	++
**Gp2 (sample 2) Pre-F**	++	**+G**	++
Gp2 (sample 3) Pre-F	+++	+G/M	+++
Gp3 (sample 1) Pre-F+CAF01	++	+++G/M	++
**Gp3 (sample 2) Pre-F+CAF01**	++	**++/G/M**	++
Gp3 (sample 3) Pre-F+CAF01	++	+G/M	+
Gp4 (sample 1) Pre-F+CAF08b	++	++G/M	++
Gp4 (sample 2) Pre-F+CAF08b	+	++G/M	+++
**Gp4 (sample 3) Pre-F+CAF08b**	++	**++G/M**	++
**Gp5 (sample 1) Pre-F+Addavax**	++	**++G/M**	++
Gp5 (sample 2) Pre-F+Addavax	+	+G/M	++
Gp5 (sample 3) Pre-F+Addavax	++	++G/M	+++
Gp6 (sample 1) Post-F+Alum	++	+G/M	+
**Gp6 (sample 2) Post-F+Alum**	++	**+G/M**	++
Gp6 (sample 3) Post-F+Alum	++	+G/M	++

Supplementary Figure 7. Pathological evaluation of RSV-infected CB6F1 mice

Immunized mice as described in Figures 4+5 were challenged with live RSV A2 strain to assess protective potential of the vaccine formulations. Weight of each animal was measured over a period of 14 days (n=5 mice/group). **(A).** Lungs from 3 animals in each immunization group were stained with H&E. PAS and EMBP for histopathological assessment of damage and immune cell infiltration. Scale bars represent 100 micrometer. **(B).** Presence of inflammatory cells (H&E), Goblet cells/Mucin (G/M) (PAS), or eosinophils (EMBP) was given a semi-quantitative score. Samples chosen for depiction of representative images (n=3 mice/group) in Panel B are indicated in bold **(C).**
