# Supplementary material for: CAF08 adjuvant enables single dose protection against respiratory syncytial virus infection in murine newborns
Source: Nat Commun. 2022 Aug 2;13:4234. doi: 10.1038/s41467-022-31709-2 (PMC9346114; doi:10.1038/s41467-022-31709-2)
Supplement: Supplementary file 3 — Description of Additional Supplementary Files [file 41467_2022_31709_MOESM3_ESM.pdf]

Description of Additional Supplementary Files

Supplementary Dataset 1
Adult (n=4) MoDCs were stimulated with **R848**, or **vehicle** control for 30 minutes. Phosphopeptides were enriched from cell lysates and quantified by mass spectrometry as described in the Methods. Peptide intensities were analyzed using Perseus. Peptide data were exported to excel to ensure reproducibility of our results. Statistical significance (Column A) was determined by unpaired two-tailed Student's t-test. A p-value (Column B) was weighed with a permutation-based fold-change (Column C) threshold cutoff (FDR 0.05 and σ=0.2, dotted lines in Supplementary Figure 1). Additional columns indicate peptide sequences and attributes.

Supplementary Dataset 2
Adult (n=4) MoDCs were stimulated with **TDB**, or **vehicle** control for 30 minutes. Phosphopeptides were enriched from cell lysates and quantified by mass spectrometry as described in the Methods. Peptide intensities were analyzed using Perseus. Peptide data were exported to excel to ensure reproducibility of our results. Statistical significance (Column A) was determined by unpaired two-tailed Student's t-test. A p-value (Column B) was weighed with a permutation-based fold-change (Column C) threshold cutoff (FDR 0.05 and σ=0.2, dotted lines in Supplementary Figure 1). Additional columns indicate peptide sequences and attributes.

Supplementary Dataset 3
Adult (n=4) MoDCs were stimulated with **R848+TDB**, or **vehicle** control for 30 minutes. Phosphopeptides were enriched from cell lysates and quantified by mass spectrometry as described in the Methods. Peptide intensities were analyzed using Perseus. Peptide data were exported to excel to ensure reproducibility of our results. Statistical significance (Column A) was determined by unpaired two-tailed Student's t-test. A p-value (Column B) was weighed with a permutation-based fold-change (Column C) threshold cutoff (FDR 0.05 and σ=0.2, dotted lines in Supplementary Figure 1). Additional columns indicate peptide sequences and attributes.

Supplementary Dataset 4
Newborn (n=9) MoDCs were stimulated with **R848**, or **vehicle** control for 30 minutes. Phosphopeptides were enriched from cell lysates and quantified by mass spectrometry as described in the Methods. Peptide intensities were analyzed using Perseus. Peptide data were exported to excel to ensure reproducibility of our results. Statistical significance (Column A) was determined by unpaired two-tailed Student's t-test. A p-value (Column B) was weighed with a permutation-based fold-change (Column C) threshold cutoff (FDR 0.05 and σ=0.2, dotted lines in Supplementary Figure 1). Additional columns indicate peptide sequences and attributes.

Supplementary Dataset 5
Newborn (n=9) MoDCs were stimulated with **TDB**, or **vehicle** control for 30 minutes. Phosphopeptides were enriched from cell lysates and quantified by mass spectrometry as described in the Methods. Peptide intensities were analyzed using Perseus. Peptide data were

exported to excel to ensure reproducibility of our results. Statistical significance (Column A) was determined by unpaired two-tailed Student's t-test. A p-value (Column B) was weighed with a permutation-based fold-change (Column C) threshold cutoff (FDR 0.05 and σ=0.2, dotted lines in Supplementary Figure 1). Additional columns indicate peptide sequences and attributes.

Supplementary Dataset 6

Newborn (n=9) MoDCs were stimulated with **R848+TDB**, or **vehicle** control for 30 minutes. Phosphopeptides were enriched from cell lysates and quantified by mass spectrometry as described in the Methods. Peptide intensities were analyzed using Perseus. Peptide data were exported to excel to ensure reproducibility of our results. Statistical significance (Column A) was determined by unpaired two-tailed Student's t-test. A p-value (Column B) was weighed with a permutation-based fold-change (Column C) threshold cutoff (FDR 0.05 and σ=0.2, dotted lines in Supplementary Figure 1). Additional columns indicate peptide sequences and attributes.
