# Supplementary material for: CAF08 adjuvant enables single dose protection against respiratory syncytial virus infection in murine newborns
Source: Nat Commun. 2022 Aug 2;13:4234. doi: 10.1038/s41467-022-31709-2 (PMC9346114; doi:10.1038/s41467-022-31709-2)
Supplement: Supplementary file 10 — Reporting Summary [file 41467_2022_31709_MOESM10_ESM.pdf]

nature research

Corresponding author(s): Simon D. van Haren, Ph.D.

Last updated by author(s): May 18, 2022

Reporting Summary

Nature Research wishes to improve the reproducibility of the work that we publish. This form provides structure for consistency and transparency in reporting. For further information on Nature Research policies, see our Editorial Policies and the Editorial Policy Checklist.

Statistics

For all statistical analyses, confirm that the following items are present in the figure legend, table legend, main text, or Methods section.

n/a	Confirmed	
☐	☒	The exact sample size (*n*) for each experimental group/condition, given as a discrete number and unit of measurement
☐	☒	A statement on whether measurements were taken from distinct samples or whether the same sample was measured repeatedly
☐	☒	The statistical test(s) used AND whether they are one- or two-sided<br>*Only common tests should be described solely by name; describe more complex techniques in the Methods section.*
☐	☒	A description of all covariates tested
☐	☒	A description of any assumptions or corrections, such as tests of normality and adjustment for multiple comparisons
☐	☒	A full description of the statistical parameters including central tendency (e.g. means) or other basic estimates (e.g. regression coefficient) AND variation (e.g. standard deviation) or associated estimates of uncertainty (e.g. confidence intervals)
☐	☒	For null hypothesis testing, the test statistic (e.g. *F*, *t*, *r*) with confidence intervals, effect sizes, degrees of freedom and *P* value noted<br>*Give P values as exact values whenever suitable.*
☒	☐	For Bayesian analysis, information on the choice of priors and Markov chain Monte Carlo settings
☒	☐	For hierarchical and complex designs, identification of the appropriate level for tests and full reporting of outcomes
☒	☐	Estimates of effect sizes (e.g. Cohen's *d*, Pearson's *r*), indicating how they were calculated

Our web collection on statistics for biologists contains articles on many of the points above.

Software and code

Policy information about availability of computer code

Data collection	No code or software was used for data collection
Data analysis	Data were analyzed and graphed using Prism for MacIntosh v. 7.0 (GraphPad Sofware).

For manuscripts utilizing custom algorithms or software that are central to the research but not yet described in published literature, software must be made available to editors and reviewers. We strongly encourage code deposition in a community repository (e.g. GitHub). See the Nature Research guidelines for submitting code & software for further information.

Data

Policy information about availability of data

All manuscripts must include a data availability statement. This statement should provide the following information, where applicable:

- Accession codes, unique identifiers, or web links for publicly available datasets
- A list of figures that have associated raw data
- A description of any restrictions on data availability

The mass spectrometry proteomics data have been deposited to the ProteomeXchange Consortium via the PRIDE 84 partner repository, https://www.ebi.ac.uk/pride/, with the dataset identifier PXD025568. Source data are provided with this paper.

Field-specific reporting

Please select the one below that is the best fit for your research. If you are not sure, read the appropriate sections before making your selection.

☒ Life sciences ☐ Behavioural & social sciences ☐ Ecological, evolutionary & environmental sciences

For a reference copy of the document with all sections, see nature.com/documents/nr-reporting-summary-flat.pdf

Life sciences study design

All studies must disclose on these points even when the disclosure is negative.

Sample size	Variable, see figure legends. Generally 4-9 for human in vitro experiments, 5-10 for mouse experiments.
Data exclusions	Mass spectrometry replicates not in compliance with our data QC were excluded from analysis as described in the manuscript.
Replication	Western blots were replicated 3x using cells from different study participants. All other data: biological replicates indicated as individual data points in each graph
Randomization	Human in vitro: cells from all participants were used in all stimulation conditions, so no randomization necessary.<br>Mouse immunizations: Littermates were assigned a treatment group sequentially, to maximize the amount of different treatment groups represented within each litter.
Blinding	Blinding was not possible, as all participants were used for all in vitro stimulation conditions (see above). Participants were de-identified to protect privacy

Reporting for specific materials, systems and methods

We require information from authors about some types of materials, experimental systems and methods used in many studies. Here, indicate whether each material, system or method listed is relevant to your study. If you are not sure if a list item applies to your research, read the appropriate section before selecting a response.

Materials & experimental systems

n/a	Involved in the study	
☒	☐	Antibodies
☒	☐	Eukaryotic cell lines
☒	☐	Palaeontology and archaeology
☐	☒	Animals and other organisms
☐	☒	Human research participants
☒	☐	Clinical data
☒	☐	Dual use research of concern

Methods

n/a	Involved in the study	
☒	☐	ChIP-seq
☐	☒	Flow cytometry
☒	☐	MRI-based neuroimaging

Animals and other organisms

Policy information about studies involving animals; ARRIVE guidelines recommended for reporting animal research

Laboratory animals	CB6F1, male and female. Newborn: day of life 4-7. Adult: 4-6 weeks old.
Wild animals	Study did not involve wild animals
Field-collected samples	Study did not include field-collected samples
Ethics oversight	Non-identifiable cord blood samples were collected with approval from the Ethics Committee of the Beth Israel Deaconess Medical Center, Boston, MA (protocol number 2011P-000118) and The Brigham & Women's Hospital, Boston, MA (protocol number 2000-P-000117). All de-identified blood samples from adult (age 18–40 years old) study participants included in the experiments were collected with approval from the Ethics Committee of Boston Children's Hospital, Boston, MA (protocol number 307-05-0223), after written informed consent was provided.<br>All experiments involving animals were approved by the Animal Care and Use Committee of Boston Children's Hospital and Harvard Medical School (protocol numbers 15-11-3011 and 16-02-3130) and studies at Statens Serum Institut were approved by the Danish governmental Animal Experiments Inspectorate (protocol number 2014-15-2934-01065)

Note that full information on the approval of the study protocol must also be provided in the manuscript.

Human research participants

Policy information about studies involving human research participants

Population characteristics	Adult participants: age 18-40, male and female. Exclusion criteria: fever or cold symptoms in the 7 days prior to blood donation.<br>Newborn participants: Age 0, male and female. Exclusion criteria: Mother with fever or cold symptoms in the 7 days prior to cesarian section.
Recruitment	Recruitment took place in BCH research facilities by oral interview by study staff or recruitment flyer. Recruitment flyer is used to gain a wider pool of study subjects. Staff did not recruit participants among under the direct supervision of the PI.
Ethics oversight	Boston Children's Hospital

Note that full information on the approval of the study protocol must also be provided in the manuscript.

Flow Cytometry

Plots

Confirm that:

☒ The axis labels state the marker and fluorochrome used (e.g. CD4-FITC).

☒ The axis scales are clearly visible. Include numbers along axes only for bottom left plot of group (a 'group' is an analysis of identical markers).

☒ All plots are contour plots with outliers or pseudocolor plots.

☒ A numerical value for number of cells or percentage (with statistics) is provided.

Methodology

Sample preparation	Cells were fixated in 1% paraformaldehyde for 1 hour at 4 degrees Celcius. For intracellular staining, cells were subsequently permeabilized with BD Perm/Wash for 30 minutes at room temperature prior to antibody staining. Cells were stained with antibodies and tetramers as indicated in the figures. 30 minutes 4C for antibodies, 1 hour 37C for tetramers. Cells were washed 3x with PBS prior to analysis
Instrument	BD LSRFortessa
Software	Acquisition: BD FACSDiva v9.0<br>Analysis: FLowjo v10.3
Cell population abundance	No sorting was done. Relevant percentages of abundant populations are indicated in the representative gating figures.
Gating strategy	FSC/SSC gates were set to be inclusive of most events except cell debris. Cells were subsequently displayed on FSC-A vs FSC-H and single cells were gates, defined a cells within a straight diagonal. All cells within this gate were used, as described in Figures 4+5.

☒ Tick this box to confirm that a figure exemplifying the gating strategy is provided in the Supplementary Information.
